# Is there a sex difference in postoperative prognosis of hepatocellular carcinoma?

**DOI:** 10.1186/s12885-019-5453-3

**Published:** 2019-03-20

**Authors:** Ming-Wei Lai, Yu-De Chu, Chih-Lang Lin, Rong-Nan Chien, Ta-Sen Yeh, Tai-Long Pan, Po-Yuan Ke, Kwang-Hui Lin, Chau-Ting Yeh

**Affiliations:** 1 Liver Research Center, Chang Gung Memorial Hospital, Taoyuan, Taiwan; 2 Division of Pediatric Gastroenterology, Department of Pediatrics, Chang Gung Memorial Hospital, Taoyuan, Taiwan; 3 Liver Research Unit, Chang Gung Memorial Hospital, Keelung, Taiwan; 4 Department of Hepato-gastroenterology, Chang Gung Memorial Hospital, Taoyuan, Taiwan; 5 Department of Surgery, Chang Gung Memorial Hospital, Taoyuan, Taiwan; 6 School of Traditional Chinese Medicine, Chang Gung University, Taoyuan, Taiwan; 7 Department of Biochemistry and Molecular Biology, Chang Gung University, Taoyuan, Taiwan; 8 Department of Biochemistry, School of Medicine, Chang-Gung University, Taoyuan, Taiwan; 9 Molecular Medicine Research Center, Chang Gung University, Taoyuan, Taiwan

**Keywords:** Hepatocellular carcinoma, Prognosis, Alpha-fetoprotein, Survival

## Abstract

**Background:**

Although men carry a higher risk of hepatocellular carcinoma (HCC) than women, it is still controversial whether men also have a poorer postoperative prognosis. A retrospective study was conducted to evaluate the postoperative prognostic predictors of HCC focusing on sex differences.

**Methods:**

We enrolled 516 consecutive adult patients with HCC (118 women, 398 men), who received surgical resection between January 2000 and December 2007, and were followed-up for >10 years. Clinical and laboratory data together with postoperative outcomes were reviewed.

**Results:**

At baseline, female patients had a higher anti-hepatitis C virus antibody prevalence (P = 0.002); lower hepatitis B virus surface antigen prevalence (P = 0.006); less microvascular invasion (P = 0.019); and lower alpha-fetoprotein (P = 0.023), bilirubin (P = 0.002), and alanine transaminase (P = 0.001) levels. Overall, there were no significant sex differences in terms of intrahepatic recurrence-free survival (RFS), distant metastasis-free survival (MFS), and overall survival (OS). However, subgroup analysis showed that women had favorable RFS (P = 0.019) and MFS (P = 0.034) in patients with alpha-fetoprotein ≤ 35 ng/mL, independent of other clinical variables (adjusted P = 0.008 and 0.043, respectively). Additionally, men had favorable OS in patients with prothrombin time (international normalized ratio [INR]) <1.1 (P = 0.033), independent of other clinical variables (adjusted P = 0.042).

**Conclusions:**

Female sex is independently associated with favorable postoperative RFS and MFS in patients with alpha-fetoprotein ≤35 ng/mL, while male sex is independently associated with favorable OS in patients with prothrombin time INR <1.1.

**Electronic supplementary material:**

The online version of this article (10.1186/s12885-019-5453-3) contains supplementary material, which is available to authorized users.

## Background

Liver cancer is ranked as the sixth most common solid cancer worldwide, with an estimated occurrence of 782,000 new cases each year. It is ranked fifth among cancers in men (554,000 cases/year) and ninth among cancers in women (228,000 cases/year). Approximately 745,000 people die of liver cancer each year, making it the second leading cause of cancer-related death. It is ranked the second deadliest cancers in men (521,000 deaths/year) and the fourth most deadly cancers in women (224,000 deaths/year) [1]. Hepatocellular carcinoma (HCC) is the most common type of primary liver cancer in adults, accounting for approximately 80% of all liver cancers. Development of HCC is largely associated with chronic hepatitis B virus (HBV) or hepatitis C virus (HCV) infections, as well as environmental toxins including aflatoxin, alcohol, and cigarette smoking [2, 3]. There is a close geographical correlation between HBV endemic areas and HCC prevalent regions, such as sub-Saharan Africa, China, Hong Kong, and Taiwan [4]. Japan has one of the highest incidence rates of HCV-associated HCC, which appears to be decreasing in recent years, while the incidence in the US has been increasing over the past two decades [5, 6].

In almost all parts of the world, men are more likely than women to develop HCC, ranging from 1- (Central America) to 4.8-fold (France) [1, 3, 7]. In the Asia-Pacific region, men are affected 1.3- (Japan) to 4.7-fold (Singapore) more frequently than women [8]. The sex disparity in the development of liver cancer is thought to be due to variations in hepatitis carrier states (more hepatitis B infections in men), follow-up/treatment compliance and exposure to environmental toxins [9, 10]. Androgen/androgen receptor signaling is known to be involved in the initiation of carcinogen-related or HBV-related HCC in men [11], whereas estrogen has been shown to exert protective effects against HCC through interleukin-6 (IL-6) restraints, STAT3 (Signal Transducer and Activator of Transcription-3) inactivation, and tumor-associated macrophage inhibition [12–15].

In Taiwan, which is an HBV endemic region, HBV surface antigen (HBsAg) was found to be positive in around 80% of male patients with HCC in the 1980s, which gradually decreased to ~70% by the late 1990s. A similar trend was also found in women [16, 17]. A multicenter cohort study enrolling 3483 patients with HCC in Taiwan between 2005 and 2011 showed that the male-to-female ratios were 6:1 in HBV-related, 2:1 in HCV-related, 3:1 in both HBV/HCV-related and 4:1 in non-B/ non-C-related HCC [18]. The cumulative lifetime incidences of HCC for men and women, who were positive for HBsAg, were significantly different (27.4% and 8%, respectively) [19].

Several systems have been proposed to predict the prognosis of HCC, which is more complex than other cancers because of the frequent coexistence of chronic liver disease. The Barcelona Clinic Liver Cancer (BCLC) staging system has shown the optimal independent predictive power of survival when compared with other prognostic systems (Okuda, Tumor-Node-Metastasis (TNM), Cancer of Liver Italian Program (CLIP), Chinese University Prognostic Index (CUPI), Japanese Integrated System (JIS), and Groupe d’Etude de Traitement du Carcinoma Hepatocellulaire (GRETCH)) [20–24]. The survival of HCC is undoubtedly affected by treatment modality, which is applied according to tumor staging [25, 26]. Notably, none of the prognostic stratification systems have proposed to separate men from women in the evaluation of HCC.

Although sex differences in HCC development risk are well recognized, the prognosis between sexes remains controversial. In a Japanese nationwide survey of 4649 HCC cases, male sex was an independent risk factor for poorer prognosis [24]. Another 12-year single-center series of 704 HCC cases in Japan found a significantly longer survival in women [27]. In an Italian survey of 600 untreated HCC cases, female sex was an independent predictor of better survival [26]. Another Italian study also showed female patients with HCC had longer survival [9]. However, some other series did not demonstrate a sex difference in HCC prognosis [21, 23]. Although female patients with HCC typically present at an older age and with lower tumor burden at diagnosis, female sex was not an independent predictor of survival in an 1886 HCC cases from an American report [28].

Due to the sex disparity in HCC incidence and controversial issues regarding sex differences in HCC prognosis, it is unclear whether postoperative surveillance and management of HCC require stratification between sexes. To clarify this issue, this retrospective study was conducted to evaluate the postoperative prognostic predictors of HCC that focused on sex differences.

## Methods

### Patients

The study protocol conformed to the ethical guidelines of the 1975 Declaration of Helsinki as reflected in a priori approval by the Institutional Review Board (201700107B0C501), Chang Gung Memorial Hospital, Taiwan. This retrospective study enrolled 516 consecutive adult patients who were diagnosed with HCC and received surgical resection at Chang Gung Memorial Hospital between January 2000 and December 2007 and had follow-up durations of up to 10 years. HCC diagnosis was confirmed by the pathologic diagnosis of surgical specimens. In our institute, all HCC patients had to be evaluated before surgery to make sure that a clean margin of > 1cm could be achieved. And thus, all our patients had an R0 status. No adjuvant anticancer treatment was given for our patients. Anti-HBV treatment (nucleos(t) ide analogue) was given to chronic hepatitis B patients with serum HBV-DNA levels > 2000 IU/L according to our National Insurance Policy.

### Clinicopathological factors evaluated

Radiology, operational findings, and pathology reports were reviewed to determine tumor characteristics, including the largest tumor size (the longest diameter), number of tumors, cirrhosis of the non-cancerous liver, histology grade of tumors (grade I to IV based on Edmondson’s grading system), branched portal vein invasion (macrovascular invasion), microvascular invasion, capsule, and ascites. Demographic information was retrieved from the charts, including sex, age, HBsAg, anti-HCV antibody, baseline laboratory data (albumin, bilirubin, prothrombin time [PT], international normalized ratio [INR], creatinine, aspartate transaminase [AST], alanine transaminase [ALT], and alpha-fetoprotein [AFP]). Alcoholism was defined as prolonged alcohol abuse leading to psychological and physical dependence.

### Statistical analysis

Continuous data that were normally distributed were reported as the mean ± standard deviation and categorical variables were expressed as number (%). Non-parametric data were shown as the median value (range). Comparison of continuous data was performed using the Student’s t-test or Mann-Whitney’s U test, where appropriate. Comparison of the categorical variables was performed by the Fisher’s exact test or Chi-square test with Yates’ correction, as appropriate. Survival analysis was evaluated by Cox proportional hazard model and verified by Kaplan-Meier analysis. Variables with a P-value <0.05 on univariate analysis were included in the multivariate analysis. Statistical comparisons for survival curves were analyzed by the log-rank test. A two-tailed *P*-value < 0.05 was considered statistically significant.

## Results

### Baseline characteristics between male and female patients with HCC

A total of 516 patients who received surgical resection for HCC were included in this study. Of them, 118 were women and 398 were men. Baseline clinical data are listed in Table 1. The comparison between female and male patients with HCC showed a significant difference in several etiologies: positive anti-HCV was found in 44 (37.3%) and 91 (22.9%) patients, respectively (P = 0.002); positive HBsAg was found in 70 (59.3%) and 289 (72.6%) patients, respectively (P = 0.006); and alcoholism was found in 3 (2.5%) and 130 (32.7%) patients, respectively. More male patients with HCC developed microvascular invasion (men vs. women, 144 [36.2%] and 29 [24.6%], P = 0.019). In laboratory data, male patients with HCC had higher AFP levels (P = 0.023); higher bilirubin levels (P = 0.002); and higher ALT levels (P = 0.001). No significant difference was found for the other parameters.

**Table 1:** Comparison between the characteristics of male and female HCCs

Clinical variables	Female (n = 118)	Male (n = 398)	P
Age	57.7 ± 14.3	56.2 ± 13.8	0.310
Anti-HCV positive, n (%)	44 (37.3%)	91 (22.9%)	**0.002**
HBsAg positive, n (%)	70 (59.3%)	289 (72.6%)	**0.006**
Liver cirrhosis, n (%)	67 (56.8%)	230 (57.8%)	0.846
Non-cirrhosis, ALT <2×ULN^a^, n (%)	43 (36.4%)	125 (31.4%)	0.361
Non-cirrhosis, ALT >2×ULN, n (%)	8 (6.8%)	43 (10.8%)	0.267
Microvascular invasion, n (%)	29 (24.6%)	144 (36.2%)	**0.019**
Macrovascular invasion, n (%)	13 (11.0%)	54 (13.6%)	0.469
Histology grade			
< 3	54 (45.8%)	179 (45.0%)	0.964
>= 3	64 (54.2%)	219 (55.0%)	
Capsule, n (%)	86 (72.9%)	289 (72.6%)	0.954
Tumor number			0.817
1	74 (62.7%)	232 (58.3%)	
2	27 (22.9%)	89 (22.4%)	
3	13 (11.0%)	54 (13.6%)	
> 3	4 (3.4%)	23 (5.8%)	
Ascites, n (%)	8 (6.8%)	31 (7.8%)	0.716
Alcoholism, n (%)	3 (2.5%)	130 (32.7%)	**< 0.001**
Largest tumor size, cm	5.9 ± 4.3	6.0 ± 5.4	0.715
AFP, ng/mL	25 (< 1 – 286980)	58.4 (1.1 – 685353)	**0.023**
Albumin, g/L	3.9 ± 0.6	4.0 ± 0.6	0.174
Bilirubin, mg/dL	0.9 ± 0.6	1.2 ± 1.4	**0.002**
Prothrombin time, sec	11.9 ± 1.4	12.2 ± 1.4	0.051
Creatinine, mg/dL	1.0 ± 1.1	1.2 ± 1.0	0.125
AST, U/L	70.0 ± 84.8	70.0 ± 99.0	0.960
ALT, U/L	53.7 ± 50.5	79.7 ± 125.3	**0.001**

^a^*ULN*, upper limit of normal; Values in bold, *P* < 0.05

### Comparison between female and male patients for postoperative prognosis including all 516 patients

**Fig. 1:**
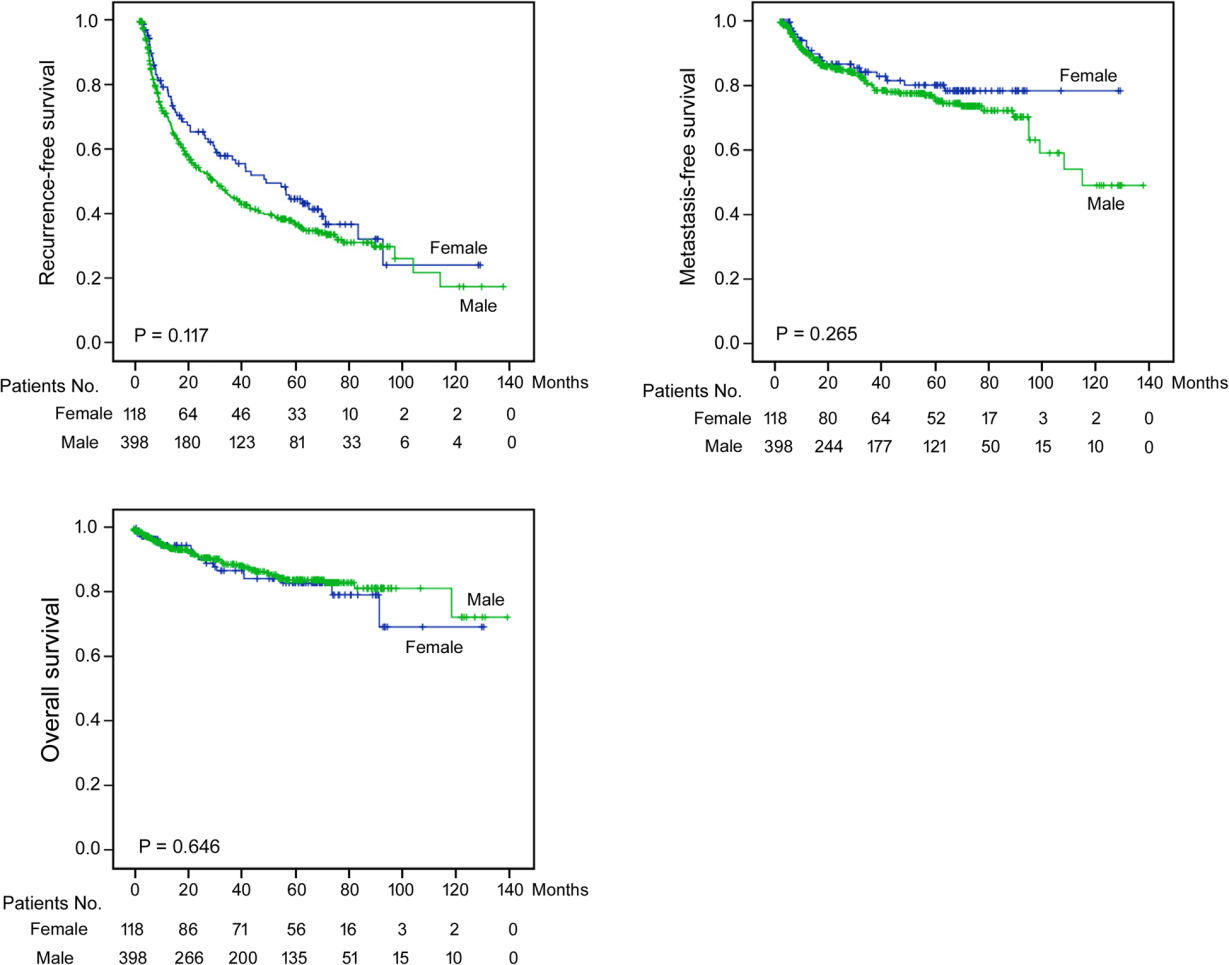
Survival differences between male and female patients included in this study. All patients were submitted for analysis. Upper left, intrahepatic recurrence-free survivals; Upper right, distant metastasis-free survivals; Lower, overall survivals. Blue curve, female; Green curve, male; The median follow-up period was 43.1 (range 1 to 139) months for all patients

Kaplan-Meier analysis was performed to compare postoperative prognosis between female and male patients (Fig. 1). No significant difference was found between the two groups when Log-rank P was calculated (recurrence-free survival: female versus male, mean (95% CI) = 59.5 (48.2 to 70.8) versus 53.1 (46.3 to 59.8) months, P = 0.117; distant metastasis-free survival: 107.3 (98.2 to 116.5) versus 100.0 (92.0 to 107.9) months, P = 0.265; overall survival: 105.8 (94.8 to 116.8) versus 117.2 (111.2 to 123.2) months, P = 0.646). However, if Breslow (Generalized Wilcoxon) P was calculated, the P values were 0.053, 0.390, and 0.826, respectively. Apparently, a borderline (but not significant) P value was found for recurrence-free survival. No patient underwent liver transplantation during the follow-up period. The 1-year, 3-year, and 5-year overall survival rates in male and female patients were about 95%, 90% and 87% without significant difference and the details including MFS and RFS were listed in Additional file 1: Table S1

### Differential prognosis predictors for male and female patients with HCC

As an attempt to identify independent risk factors predictive of postoperative survival or recurrence among male and female patients with HCC and their potential differences, we conducted the analysis separately in male and female patients using the Cox proportional hazard analysis (Additional files 2, 3 and 4: Table S2, S3 and S4). After univariate and multivariate analyses, it was found that male and female patients with HCC had different sets of independent predictors. The significant independent predictors of intrahepatic recurrence-free survival were microvascular invasion (P <0.001), tumor number (P = 0.039), albumin levels (P = 0.004), and AST levels (P = 0.025) for men; for women, the predictors were microvascular invasion (P = 0.002) and AST levels (P < 0.001) (Additional file 2: Table S2). The predictors of metastasis-free survival were microvascular invasion (P <0.001), macrovascular invasion (P = 0.002), and AFP levels (P = 0.032) for men; for women, the predictors were AFP levels (P = 0.002) and AST levels (P = 0.001) (Additional file 3: Table S3). The predictors of overall survival were tumor number (P = 0.019) and albumin levels (P = 0.045) for men, and only bilirubin levels (P = 0.008) for women (Additional file 4: Table S4).

### Survival and AFP levels

Subsequently, we performed subgroup analysis to identify subgroups wherein there was a sex difference in term of postoperative prognosis. When intrahepatic recurrence-free survival was compared between all female and male patients with HCC by Cox proportional hazard model, it was found that there was no significant difference (Table 2; P = 0.118). Subgroup analysis was performed to investigate whether one or more of the subgroups displayed sex difference in terms of recurrence-free survival (Table 2). Median values were used as cut-offs for all continuous variables. It was found that in two subgroups, female patients had a favorable recurrence-free survival: patients with ascites (n = 39; P = 0.019) and patients with AFP ≤35 ng/mL (n = 270; P = 0.019). None of the other subgroups showed significant sex differences in terms of recurrence-free survival. When we analyzed the subgroup with AFP ≤35 ng/mL (n = 270), univariate and multivariate analyses both showed sex was an independent predictor of recurrence-free survival (Table 3). When distant metastasis-free survival was compared, it was found that there was no difference between sexes (Table 4; P = 0.267). However, subgroup analysis showed favorable metastasis-free survival in female patients with lower AFP ≤35 ng/mL (n = 270; P = 0.034). None of the other subgroups showed sex differences in terms of distant metastasis-free survival. When we analyzed the subgroup with AFP ≤35 ng/mL (n = 270), sex remained an independent determinant of metastasis-free survival in both univariate and multivariate analyses (Table 5).

**Table 2:** Cox proportional hazard analysis for sex difference in relationship to intrahepatic recurrence-free survival in various clinical subgroups (Male = 1)

		No. of patients	HR	95% CI	P
Overall		516	1.255	0.944 – 1.668	0.118
Age, years	<58	262	1.448	0.912 – 2.298	0.117
	≥58	254	1.162	0.801 – 1.685	0.430
Anti-HCV	Negative	381	1.329	0.928 – 1.902	0.121
	Positive	135	1.280	0.779 – 2.103	0.330
HBsAg	Negative	157	1.591	0.983 – 2.575	0.059
	Positive	359	1.118	0.783 – 1.598	0.539
Liver cirrhosis	No	219	1.533	0.955 – 2.460	0.077
	Yes	297	1.080	0.754 – 1.545	0.675
Microvascular invasion	No	343	1.090	0.772 – 1.539	0.625
	Yes	173	1.203	0.710 – 2.038	0.491
Macrovascular invasion	No	449	1.283	0.943 – 1.747	0.113
	Yes	67	0.939	0.442 – 1.996	0.870
Histology grade	<3	233	1.477	0.938 – 2.327	0.092
	≥3	283	1.123	0.778 – 1.621	0.534
Capsule	No	141	1.328	0.766 – 2.303	0.312
	Yes	375	1.219	0.873 – 1.700	0.245
Tumor number	1	306	1.170	0.806 – 1.697	0.409
	>1	210	1.295	0.830 – 2.022	0.255
Ascites	No	477	1.133	0.846 – 1.516	0.403
	Yes	39	5.798	1.336 – 25.168	**0.019**
Alcoholism	No	383	1.227	0.905 – 1.665	0.188
	Yes	133	0.685	0.168 – 2.796	0.598
Largest tumor size, cm	≤4	242	1.176	0.784 – 1.764	0.434
	>4	274	1.276	0.853 – 1.910	0.236
AFP, ng/mL	≤35	270	1.796	1.102 – 2.926	**0.019**
	>35	246	1.085	0.760 – 1.550	0.653
Albumin, g/L	≤4	279	1.372	0.937 – 2.008	0.104
	>4	237	1.139	0.741 – 1.752	0.552
Bilirubin, mg/dL	≤0.8	263	1.336	0.913 – 1.955	0.136
	>0.8	253	1.100	0.713 – 1.696	0.668
PT, sec	<12	259	1.200	0.797 – 1.808	0.382
	≥12	257	1.264	0.848 – 1.885	0.250
PT, INR	<1.1	270	1.230	0.819 – 1.846	0.319
	≥1.1	246	1.261	0.844 – 1.884	0.258
Creatinine, mg/dL	≤1	294	1.256	0.906 – 1.739	0.171
	>1	222	1.342	0.625 – 2.882	0.450
AST, U/L	≤39	262	1.351	0.865 – 2.110	0.186
	>39	254	1.206	0.832 – 1.747	0.323
ALT, U/L	≤40	255	1.493	0.989 – 2.260	0.058
	>40	261	0.988	0.667 – 1.464	0.954

*AFP* alpha-fetoprotein, *PT* prothrombin time, *INR* international normalized ratio, *AST* aspartate aminotransferase, *ALT* alanine aminotransferase, *HR* hazard ratio, *CI* confidence interval; Values in bold, *P* < 0.05

**Table 3:** Cox proportional hazard analysis for clinical variables in relationship to recurrence-free survival in AFP ≤ 35 ng/mL subgroup (n = 270)

	HR	95% CI	P
Univariate analysis			
Age, per year	1.013	0.999 – 1.027	0.070
Gender, Male = 1	1.796	1.102 – 2.926	**0.019**
Anti-HCV, positive = 1	1.299	0.882 – 1.915	0.186
HBsAg, positive = 1	0.867	0.597 – 1.259	0.454
Liver cirrhosis, yes = 1	1.379	0.961 – 1.979	0.081
Microvascular invasion, yes = 1	2.285	1.582 – 3.302	**< 0.001**
Macrovascular invasion, yes = 1	2.003	1.181 – 3.398	**0.010**
Histology grade, per grade	1.340	1.035 – 1.735	**0.026**
Capsule, yes = 1	1.040	0.709 – 1.526	0.840
Tumor number, per number	1.207	1.021 – 1.427	**0.027**
Ascites yes = 1	2.309	1.169 – 4.561	**0.016**
Alcoholism yes = 1	1.170	0.799 – 1.713	0.419
Largest tumor size, per cm	0.997	0.970 – 1.025	0.838
AFP, per ng/mL	1.034	1.014 – 1.055	**0.001**
Albumin, per g/L	0.642	0.431 – 0.857	**0.003**
Bilirubin, per mg/dL	0.919	0.765 – 1.105	0.371
PT, per sec	1.074	0.966 – 1.195	0.187
Creatinine, per mg/dL	1.039	0.884 – 1.220	0.645
AST, per U/L	1.003	1.001 – 1.004	**0.001**
ALT, per U/L	1.002	1.000 – 1.003	**0.011**
Multivariate analysis			
Gender, Male = 1	1.997	1.198 – 3.327	**0.008**
Microvascular invasion, yes = 1	2.057	1.381 – 3.062	**< 0.001**
Macrovascular invasion, yes = 1	1.580	0.880 – 2.837	0.126
Histology grade, per grade	1.075	0.816 – 1.417	0.606
Tumor number, per number	1.137	0.948 – 1.362	0.166
Ascites yes = 1	1.566	0.751 – 3.265	0.231
AFP, per ng/mL	1.032	1.011 – 1.054	**0.003**
Albumin, per g/L	0.709	0.521 – 0.964	**0.028**
AST, per U/L	1.002	0.998 – 1.006	0.257
ALT, per U/L	1.000	0.997 – 1.003	0.999

*AFP* alpha-fetoprotein, *PT* prothrombin time, *AST* aspartate aminotransferase, *ALT* alanine aminotransferase, *HR* hazard ratio, *CI* confidence interval; Values in bold, *P* < 0.05

**Table 4:** Cox proportional hazard analysis for sex difference in relationship to metastasis-free survival in various clinical subgroups (Male = 1)

		No. of patients	HR	95% CI	P
Overall		516	1.328	0.805 – 2.193	0.267
Age, years	<58	262	0.957	0.496 – 1.847	0.896
	≥58	254	1.731	0.793 – 3.779	0.169
Anti-HCV	Negative	381	1.272	0.701 – 2.307	0.429
	Positive	135	1.369	0.523 – 3.583	0.522
HBsAg	Negative	157	2.159	0.876 – 5.321	0.094
	Positive	359	1.049	0.574 – 1.918	0.877
Liver cirrhosis	No	219	1.272	0.615 – 2.632	0.517
	Yes	297	1.373	0.688 – 2.742	0.368
Microvascular invasion	No	343	1.024	0.541 – 1.937	0.943
	Yes	173	1.324	0.562 – 3.119	0.521
Macrovascular invasion	No	449	1.331	0.758 – 2.340	0.320
	Yes	67	1.147	0.379 – 3.477	0.808
Histology grade	<3	233	2.094	0.816 – 5.370	0.124
	≥3	283	1.076	0.592 – 1.958	0.809
Capsule	No	141	0.868	0.366 – 2.056	0.747
	Yes	375	1.550	0.831 – 2.890	0.168
Tumor number	1	306	1.242	0.639 – 2.415	0.523
	>1	210	1.362	0.631 – 2.940	0.431
Ascites	No	477	1.246	0.753 – 2.061	0.392
	Yes	39	31.863	0.002 - 458965	0.479
Alcoholism	No	383	1.248	0.736 – 2.116	0.411
	Yes	133	20.884	0.000 - 1274875	0.589
Largest tumor size, cm	≤4	242	1.851	0.769 – 4.456	0.169
	>4	274	1.060	0.575 – 1.952	0.852
AFP, ng/mL	≤35	270	3.572	1.104 – 11.553	**0.034**
	>35	246	0.956	0.534 – 1.714	0.881
Albumin, g/L	≤4	279	1.378	0.728 – 2.606	0.325
	>4	237	1.356	0.601 – 3.061	0.463
Bilirubin, mg/dL	≤0.8	263	1.418	0.746 – 2.694	0.287
	>0.8	253	1.154	0.511 – 2.604	0.730
PT, sec	<12	259	1.105	0.559 – 2.184	0.774
	≥12	257	1.572	0.740 – 3.340	0.239
PT, INR	<1.1	270	1.189	0.607 – 2.328	0.614
	≥1.1	246	1.496	0.700 – 3.195	0.299
Creatinine, mg/dL	≤1	294	1.325	0.776 – 2.265	0.303
	>1	222	22.955	0.098 - 5387	0.261
AST, U/L	≤39	262	1.124	0.538 – 2.351	0.756
	>39	254	1.511	0.761 – 3.000	0.238
ALT, U/L	≤40	255	1.695	0.849 – 3.384	0.134
	>40	261	1.040	0.503 – 2.153	0.915

*AFP* alpha-fetoprotein, *PT* prothrombin time, *INR* international normalized ratio, *AST* aspartate aminotransferase, *ALT* alanine aminotransferase, *HR* hazard ratio, *CI* confidence interval; Values in bold, *P* < 0.05

**Table 5:** Cox proportional hazard analysis for clinical variables in relationship to metastasis-free survival in AFP ≤ 35 ng/mL subgroup (n = 270)

	HR	95% CI	P
Univariate analysis			
Age, per year	1.017	0.992 – 1.042	0.188
Gender, Male = 1	3.572	1.104 – 11.553	**0.034**
Anti-HCV, positive = 1	1.208	0.618 – 2.360	0.581
HBsAg, positive = 1	0.866	0.456 – 1.643	0.660
Liver cirrhosis, yes = 1	1.125	0.610 – 2.074	0.707
Microvascular invasion, yes = 1	2.742	1.491 – 5.041	**0.001**
Macrovascular invasion, yes = 1	4.081	1.999 – 8.332	**< 0.001**
Histology grade, per grade	1.473	0.951 – 2.281	0.083
Capsule, yes = 1	0.980	0.511- 1.880	0.952
Tumor number, per number	1.308	1.007 – 1.700	**0.044**
Ascites yes = 1	1.048	0.253 – 4.348	0.948
Alcoholism yes = 1	1.043	0.535 – 2.033	0.9903
Largest tumor size, per cm	1.009	0.973 – 1.047	0.613
AFP, per ng/mL	1.037	1.003 – 1.073	**0.035**
Albumin, per g/L	0.740	0.442 – 1.239	0.252
Bilirubin, per mg/dL	0.954	0.713 – 1.275	0.748
PT, per sec	1.084	0.903 – 1.302	0.385
Creatinine, per mg/dL	0.891	0.567 – 1.399	0.615
AST, per U/L	1.001	0.998 – 1.004	0.534
ALT, per U/L	1.000	0.996 – 1.003	0.787
Multivariate analysis			
Gender, Male = 1	3.413	1.042 – 11.183	**0.043**
Microvascular invasion, yes = 1	2.687	1.426 – 5.060	**0.002**
Macrovascular invasion, yes = 1	4.209	1.867 – 9.490	**0.001**
Tumor number, per number	1.004	0.749 – 1.347	0.977
AFP, per ng/mL	1.052	1.015 – 1.089	**0.005**

*AFP* alpha-fetoprotein, *PT* prothrombin time, *AST* aspartate aminotransferase, *ALT* alanine aminotransferase, *HR* hazard ratio, *CI* confidence interval; Values in bold, *P* < 0.05

### Overall survival and PT

**Fig. 2:**
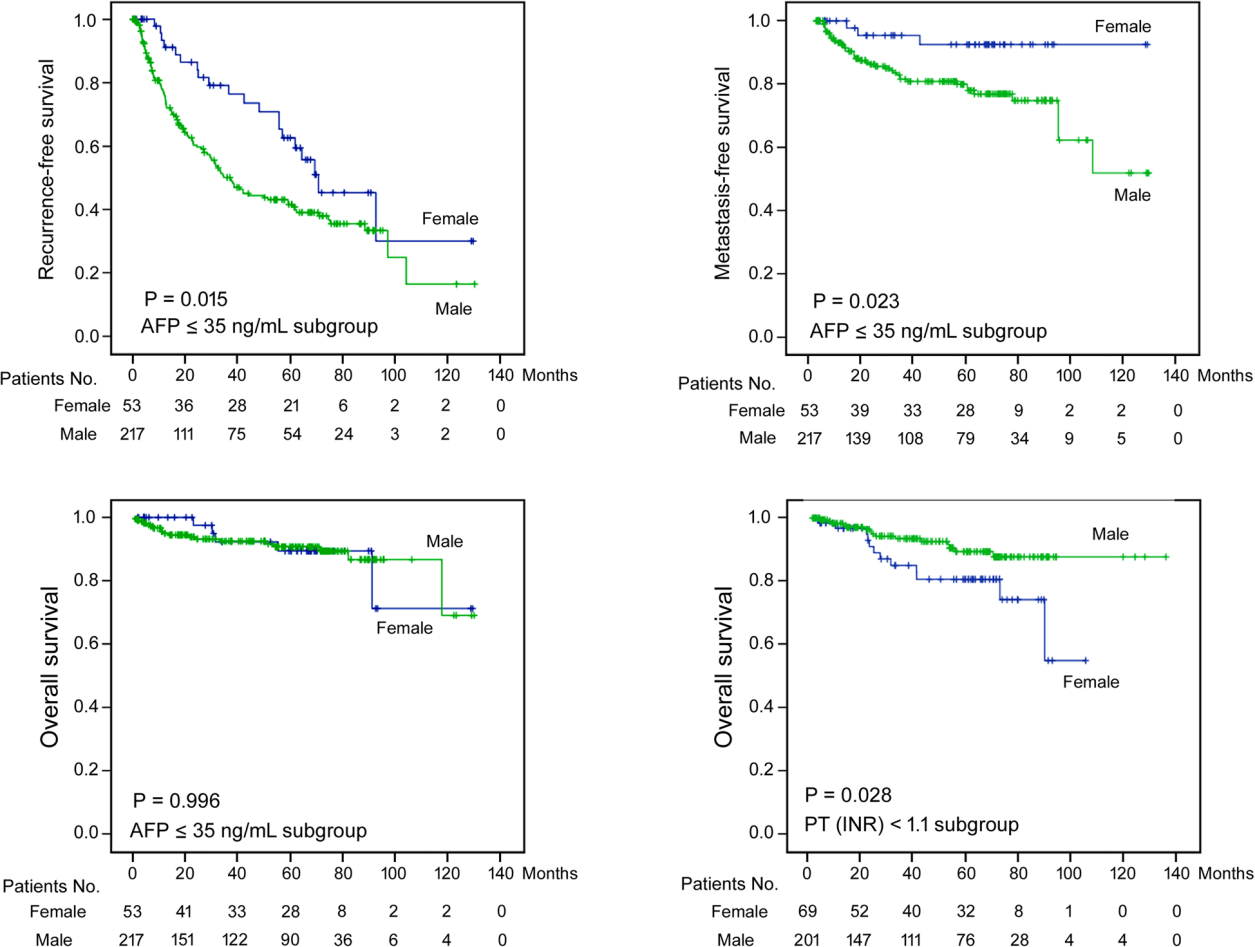
Sex differences in postoperative prognoses of patient subgroups. Kaplan-Meier analysis was performed on 270 patients with AFP ≤35 ng/mL (Upper left, intrahepatic recurrence-free survival; Upper right, metastasis-free survival; Lower left, overall survival). Kaplan-Meier analysis was also performed on 270 patients with PT INR <1.1 (Lower right, overall survival). Blue curve, women; Green curve, men

Subsequently, we analyzed sex differences in relation to overall survival in various clinical subgroups. Again, it was found that there was no sex difference, in terms of overall survival, when all patients were compared (Table 6; P = 0.646). Intriguingly, when subgroup analysis was performed, the subgroup with lower AFP did not show a sex difference (P = 0.923). In contrast, for the subgroups with shorter PT <12 sec (n = 259) or INR <1.1 (n = 270), male but not female patients with HCC showed a favorable overall survival (P = 0.042 and 0.033, respectively). Kaplan-Meier analysis also supported this finding (Fig. 2**)** When we analyzed the subgroup with INR <1.1 (n=270), sex remained an independent determinant of overall survival in both univariate and multivariate analyses (Table 7).

**Table 6:** Cox proportional hazard analysis for sex difference in relationship to overall survival in various clinical subgroups (Male = 1)

		No. of patients	HR	95% CI	P
Overall		516	0.881	0.513 – 1.513	0.646
Age, years	<58	262	1.671	0.590 – 4.735	0.334
	≥58	254	0.567	0.279 – 1.152	0.117
Anti-HCV	Negative	381	0.892	0.468 – 1.701	0.729
	Positive	135	0.813	0.288 – 2.291	0.695
HBsAg	Negative	157	0.556	0.236 – 1.309	0.179
	Positive	359	1.244	0.580 – 2.669	0.575
Liver cirrhosis	No	219	1.573	0.602 – 4.111	0.356
	Yes	297	0.625	0.318 – 1.228	0.172
Microvascular invasion	No	343	0.830	0.420 – 1.641	0.593
	Yes	173	0.751	0.304 – 1.854	0.535
Macrovascular invasion	No	449	0.924	0.502 – 1.699	0.798
	Yes	67	0.629	0.189 – 2.102	0.452
Histology grade	<3	233	1.005	0.403 – 2.509	0.991
	≥3	283	0.831	0.424 – 1.630	0.590
Capsule	No	141	0.621	0.231 – 1.617	0.322
	Yes	375	0.987	0.512 – 1.903	0.970
Tumor number	1	306	0.671	0.344 – 1.306	0.240
	>1	210	1.349	0.512 – 3.555	0.545
Ascites	No	477	0.880	0.487 – 1.591	0.672
	Yes	39	0.965	0.254 – 3.662	0.958
Alcoholism	No	383	0.768	0.426 – 1.385	0.381
	Yes	133	20.814	0.000 - 29575628	0.675
Largest tumor size, cm	≤4	242	0.609	0.252 – 1.470	0.270
	>4	274	1.023	0.507 – 2.062	0.950
AFP, ng/mL	≤35	270	1.003	0.374 – 2.689	0.996
	>35	246	0.968	0.504 – 1.858	0.923
Albumin, g/L	≤4	279	1.064	0.519 – 2.180	0.866
	>4	237	0.658	0.288 – 1.504	0.321
Bilirubin, mg/dL	≤0.8	263	0.674	0.327 – 1.390	0.285
	>0.8	253	1.129	0.469 – 2.721	0.786
PT, sec	<12	259	0.455	0.213 – 0.972	**0.042**
	≥12	257	1.61	0.671 – 3.819	0.289
PT, INR	<1.1	270	0.438	0.205 – 0.936	**0.033**
	≥1.1	246	1.677	0.703 – 4.001	0.243
Creatinine, mg/dL	≤1	294	1.122	0.590 – 2.136	0.725
	>1	222	0.445	0.152 – 1.301	0.139
AST, U/L	≤39	262	0.836	0.370 – 1.889	0.667
	>39	254	0.931	0.452 – 1.920	0.847
ALT, U/L	≤40	255	0.790	0.390 – 1.597	0.511
	>40	261	1.092	0.451 – 2.647	0.845

*AFP* alpha-fetoprotein, *PT* prothrombin time, *INR* international normalized ratio, *AST* aspartate aminotransferase, *ALT* alanine aminotransferase, *HR* hazard ratio, *CI* confidence interval; Values in bold, *P* < 0.05

**Table 7:** Cox proportional hazard analysis for clinical variables in relationship to overall survival in INR < 1.1 subgroup (n = 270)

	HR	95% CI	P
Univariate analysis			
Age, per year	1.003	0.977 – 1.029	0.840
Gender, Male = 1	0.438	0.205 – 0.936	**0.033**
Anti-HCV, positive = 1	1.277	0.540 – 3.021	0.578
HBsAg, positive = 1	0.501	0.232 – 1.081	0.078
Liver cirrhosis, yes = 1	1.112	0.522 – 2.366	0.783
Microvascular invasion, yes = 1	1.775	0.812 – 3.884	0.151
Macrovascular invasion, yes = 1	1.385	0.327 – 5.870	0.659
Histology grade, per grade	0.837	0.461 – 1.521	0.560
Capsule, yes = 1	0.974	0.411 – 2.309	0.953
Tumor number, per number	0.998	0.653 – 1.525	0.994
Ascites yes = 1	3.198	0.960 – 10.654	0.058
Alcoholism yes = 1	1.386	0.606 – 3.171	0.440
Largest tumor size, per cm	1.077	0.994 – 1.168	0.071
AFP, per 1000 ng/mL	1.002	0.998 – 1.006	0.346
Albumin, per g/L	0.441	0.208 – 0.937	**0.033**
Bilirubin, per mg/dL	1.725	1.086 – 2.740	**0.021**
PT, per sec	0.695	0.374 – 1.293	0.251
Creatinine, per mg/dL	0.967	0.709 – 1.320	0.834
AST, per U/L	1.003	0.999 – 1.006	0.193
ALT, per U/L	1.000	0.995 – 1.005	0.997
Multivariate analysis			
Gender, Male = 1	0.441	0.201 – 0.971	**0.042**
Albumin, per g/dL	0.560	0.270 – 1.160	0.119
Bilirubin, per mg/dL	1.802	1.131 – 2.870	**0.013**

*INR* international normalized ratio, *AFP* alpha-fetoprotein, *PT* prothrombin time, *AST* aspartate aminotransferase, *ALT* alanine aminotransferase, *HR* hazard ratio, *CI* confidence interval; Values in bold, *P* < 0.05

## Discussion

Women with resectable HCC have different postoperative prognostic predictors from men and have better recurrence-free and metastasis-free survival than men if baseline AFP < 35 ng/mL from this study. Currently, sex is not considered in diverse prognostic staging systems for HCC. This retrospective, long-term postoperative study intended to clarify this issue. The baseline characteristics showed significantly more HCV infections, but less HBV infections and alcoholism in women with HCC, which is the same etiologic spectrum as previous literature, especially in Asian populations, except the Japanese [9, 28]. Higher baseline AFP, ALT, and bilirubin levels, as well as microvascular invasion, were also characteristics of men with HCC, which indicates more aggressive tumor behavior, background hepatic necro-inflammation, and poor liver reserves; this would be expected to affect long-term, post-operative prognoses. However, there was no significant difference in intrahepatic recurrence-free survival or distant metastasis-free survival when all female and male patients were compared. After stratification for various clinical parameters, female patients with HCC showed better recurrence-free and metastasis-free survival in those with AFP ≤ 35 ng/mL or those with ascites. AFP is an important biomarker in predicting HCC outcome and is incorporated into several staging systems. However, the recommended cut-off levels of AFP vary. In CLIP, the cut-off level is 400 ng/mL (score 0, 1); in GRETCH, 35 ng/mL (score 0, 2); and in CUPI, 500 ng/mL (score 2) [29–31]. An AFP staging system sets levels of 10-150, 150-500, and >500 ng/mL for the discrimination of survival, especially in non-cirrhotic cases [32]. Lower AFP levels indicate either more favorable tumor characteristics (microvascular invasion, differentiation), less tumor burden, or non-cirrhotic background, which all imply an early HCC stage. At an early stage of HCC, female sex hormones may exert a protective role, whereas androgen may exert initiation/ promotion effects on the tumor during this phase. In patients with higher AFP, the growth regulatory effects from other signaling pathways, such as tyrosine kinase receptor-related pathways, might play a more important role that masks the effects from sex hormones.

Ascites indicates either poor liver reserves, portal hypertension, or portal vein thrombosis, which are all predictors of poor prognosis and is represented as an individual factor (Okuda, CUPI, Advanced Liver Cancer Prognostic System) or Child-Turcotte-Pugh scores in several staging systems (CLIP, BCLC, JIS, etc.) [33, 34]. However, in this operable cohort, only 39 patients presented with ascites. Better prognosis in women than men needs further validation.

Regarding overall survival, the sex analysis paradoxically favors male patients with HCC in the subgroup with good coagulation profiles (PT or INR). The reason for this seemingly contradictory observation is unclear. A possible explanation is that men, usually physically stronger, may withstand repetitive therapies, such as transarterial chemoembolization (TACE) for recurrent tumors over a long duration. Alternatively, androgen has dual but opposite effects on hepatocarcinogenesis: initiation and promotion at an early stage, whereas suppression of metastasis at a late stage, which may explain the longer overall survival in men with good liver reserves [35].

When the clinical features were separately analyzed in male and female patients with HCC, it is intriguing to discover that for different endpoints (recurrence-free survival, metastasis-free survival or overall survival), different or additional clinical features accounted for HCC outcome in male patients. This reflects the fact that sex itself may exert certain biological effects on the natural course of HCC.

HCC is a sexual dimorphic cancer with male predilection, not only in humans but also in rodents. Sex hormones are expected to play a central role in the sexual disparity of this malignancy. Li et al. found that androgen/ androgen receptor (AR) signaling mediated promotion, as well as estrogen/ estrogen receptor (ER) signaling mediated protection, of HCC, are driven by the Foxa1/Foxa2-dependent recruitment of ER-α and AR to target genes in a chemical-induced hepatocarcinogenesis mouse model. Foxa2 nucleotide polymorphisms may affect ER-α binding and correlate with the development of HCC in women [36]. Yang et al. demonstrated that estrogen reduces hepatocarcinogenesis through suppressing the alternative activation of macrophages (M2) via binding to ER-β, hence inhibiting JAK1-STAT6 signaling [15]. The correlation of lower risk and better survival of HCC with longer estrogen exposure in adult women (less parous, delayed menopause, hormone replacement therapy) has been proved in epidemiology studies in different populations [37–39]. Although animal models and epidemiology studies showed that sex hormones are determinants for the development and outcome of HCC, sex hormone-targeted therapies in HCC did not show a significant benefit over best supportive care [40, 41]. The inconsistency in these results is attributed to inappropriate selection of patients with differential expression of receptors or variant receptors. Furthermore, ER-α66 (wild-type) and ER-α36 (splicing variant) were expressed inversely in non-tumor, non-cirrhotic to cirrhotic and cancerous stages [42], which enabled ER-α wild-type or variant transcripts in the tumor to be a better staging system for discriminating HCC prognosis than other scoring systems [43]. To understand the mechanism why female sex is associated with favorable postoperative outcome in HCC patients, it is essential to examine the estrogen and androgen levels before and after operation for all patients. It is possible that the sex hormone levels have a direct regulatory effect on HCC growth, or alternatively, host cells altered by long-term sex hormone stimulations could indirectly change the properties of cancer cell growth. In this retrospectively study, however, we were unable to measure the sex hormone levels. Besides, the average age of women with operable HCC in this study was 57.7 of age, indicating most of them were in menopause and thus received less influence from estrogen, which could partly explain the similar postoperative prognosis between males and females.

## Conclusions

In this retrospective cohort of patients with surgically resectable HCC, although no significant sex differences were found in OS, RFS and MFS, we found different prognosis between male and female patients, restricted to certain subgroups (in patients with lower AFP ≤35 ng/mL, women showed better recurrence-free and metastasis-free survival; in patients with PT <1.1, men showed better overall survival). The molecular mechanisms underlying this disparity may include interactions between sex hormone-related pathways and other growth-related signaling pathways at different stages of HCC.

## Additional files

Additional file 1: **Table S1**. Sex specific 1-, 3-, and 5-year survival rates. The table lists 1-, 3-, and 5-year overall survival, metastasis-free survival, and recurrence-free survival rates in HCC patients of different sexes. (PDF 79 kb)

Additional file 2: **Table S2**. Clinicopathological factors associated with intrahepatic recurrence free survival in male and female HCC. The table lists univariate and multivariate analysis of clinicopathological factors associated with intrahepatic recurrence-free survival in HCC patients of different sexes. (PDF 91 kb)

Additional file 3: **Table** **S3**. Clinicopathological factors associated with distant metastasis free survival in male and female HCC. The table lists univariate and multivariate analysis of clinicopathological factors associated with distant metastasis free survival in HCC patients of different sexes, (PDF 91 kb)

Additional file 4: **Table** **S4**. Clinicopathological factors associated with overall survival in male and female HCC. The table lists univariate and multivariate analysis of clinicopathological factors associated with overall survival in HCC patients of different sexes. (PDF 91 kb)

## Abbreviations

AFP: Alpha-fetoprotein
ALT: Alanine transaminase
AR: Androgen receptor
AST: Aspartate transaminase
BCLC: Barcelona Clinic Liver Cancer
CLIP: Cancer of Liver Italian Program
CUPI: Chinese University Prognostic Index
ER: Estrogen receptor
GRETCH: Groupe d’Etude de Traitement du Carcinoma Hepatocellulaire
HBV: Hepatitis B virus
HCC: Hepatocellular carcinoma
HCV: Hepatitis C viru
HBsAg: HBV surface antigen
INR: International normalized ratio
JIS: Japanese Integrated System
PT: Prothrombin time
TACE: Transarterial chemoembolization
TNM: Tumor-Node-Metastasis

## References

[1] Ferlay J, Soerjomataram I, Dikshit R, Eser S, Mathers C, Rebelo M, Parkin DM, Forman D, Bray F (2015). Cancer incidence and mortality worldwide: Sources, methods and major patterns in GLOBOCAN 2012. Int J Cancer.

[2] Knudsen ES, Gopal P, Singal AG (2014). The changing landscape of hepatocellular carcinoma: etiology, genetics, and therapy. Am J Pathol.

[3] El-Serag HB (2012). Epidemiology of viral hepatitis and hepatocellular carcinoma. Gastroenterology.

[4] McGlynn KA, London WT (2011). The global epidemiology of hepatocellular carcinoma, present and future. Clin Liver Dis.

[5] Tanaka H, Imai Y, Hiramatsu N, Ito Y, Imanaka K, Oshita M, Hijioka T, Katayama K, Yabuuchi I, Yoshihara H (2008). Declining incidence of hepatocellular carcinoma in Osaka, Japan, from 1990 to 2003. Ann Intern Med.

[6] Davila JA, Morgan RO, Shaib Y, McGlynn KA, El-Serag HB (2004). Hepatitis C infection and the increasing incidence of hepatocellular carcinoma: a population-based study. Gastroenterology.

[7] Jemal A, Bray F, Center MM, Ferlay J, Ward E, Forman D (2011). Global cancer statistics. CA Cancer J Clin.

[8] Yuen MF, Hou JL, Chutaputti A (2009). Hepatocellular carcinoma in the Asia pacific region. J Gastroenterol Hepatol.

[9] Farinati F, Sergio A, Giacomin A, Di Nolfo MA, Del Poggio P, Benvegnù L, Rapaccini G, Zoli M, Borzio F, Giannini EG (2009). Is female sex a significant favorable prognostic factor in hepatocellular carcinoma?. Eur J Gastroenterol Hepatol.

[10] Hefaiedh R, Ennaifer R, Romdhane H, Ben Nejma H, Arfa N, Belhadj N, Gharbi L, Khalfallah T (2013). Gender difference in patients with hepatocellular carcinoma. Tunis Med.

[11] Jiang L, Shan J, Shen J, Wang Y, Yan P, Liu L, Zhao W, Xu Y, Zhu W, Su L (2016). Androgen/androgen receptor axis maintains and promotes cancer cell stemness through direct activation of Nanog transcription in hepatocellular carcinoma. Oncotarget.

[12] Naugler WE, Sakurai T, Kim S, Maeda S, Kim K, Elsharkawy AM, Karin M (2007). Gender disparity in liver cancer due to sex differences in MyD88-dependent IL-6 production. Science.

[13] Nakagawa H, Maeda S, Yoshida H, Tateishi R, Masuzaki R, Ohki T, Hayakawa Y, Kinoshita H, Yamakado M, Kato N (2009). Serum IL-6 levels and the risk for hepatocarcinogenesis in chronic hepatitis C patients: an analysis based on gender differences. Int J Cancer.

[14] Hou J, Xu J, Jiang R, Wang Y, Chen C, Deng L, Huang X, Wang X, Sun B (2013). Estrogen-sensitive PTPRO expression represses hepatocellular carcinoma progression by control of STAT3. Hepatology.

[15] Yang W, Lu Y, Xu Y, Xu L, Zheng W, Wu Y, Li L, Shen P (2012). Estrogen represses hepatocellular carcinoma (HCC) growth via inhibiting alternative activation of tumor-associated macrophages (TAMs). J Biol Chem.

[16] Chen DS (2007). Hepatocellular carcinoma in Taiwan. Hepatol Res.

[17] Lee CM, Hung CH, Lu SN, Wang JH, Tung HD, Huang WS, Chen CL, Chen WJ, Changchien CS (2006). Viral etiology of hepatocellular carcinoma and HCV genotypes in Taiwan. Intervirology.

[18] Chang I-C, Huang S-F, Chen P-J, Chen C-L, Chen C-L, Wu C-C, Tsai C-C, Lee P-H, Chen M-F, Lee C-M (2016). The Hepatitis Viral Status in Patients With Hepatocellular Carcinoma: a Study of 3843 Patients From Taiwan Liver Cancer Network. Medicine.

[19] Huang Y-T, Jen C-L, Yang H-I, Lee M-H, Su J, Lu S-N, Iloeje UH, Chen C-J (2011). Lifetime risk and sex difference of hepatocellular carcinoma among patients with chronic hepatitis B and C. J Clin Oncol.

[20] Marrero JA, Fontana RJ, Barrat A, Askari F, Conjeevaram HS, Su GL, Lok AS (2005). Prognosis of hepatocellular carcinoma: comparison of 7 staging systems in an American cohort. Hepatology.

[21] Grieco A, Pompili M, Caminiti G, Miele L, Covino M, Alfei B, Rapaccini GL, Gasbarrini G (2005). Prognostic factors for survival in patients with early-intermediate hepatocellular carcinoma undergoing non-surgical therapy: comparison of Okuda, CLIP, and BCLC staging systems in a single Italian centre. Gut.

[22] Cillo U, Vitale A, Grigoletto F, Farinati F, Brolese A, Zanus G, Neri D, Boccagni P, Srsen N, D'Amico F (2006). Prospective validation of the Barcelona Clinic Liver Cancer staging system. J Hepatol.

[23] Gomaa AI, Hashim MS, Waked I (2014). Comparing staging systems for predicting prognosis and survival in patients with hepatocellular carcinoma in Egypt. PLoS One.

[24] Kitai S, Kudo M, Izumi N, Kaneko S, Ku Y, Kokudo N, Sakamoto M, Takayama T, Nakashima O, Kadoya M (2014). Validation of three staging systems for hepatocellular carcinoma (JIS Score, Biomarker-Combined JIS Score and BCLC System) in 4,649 cases from a Japanese nationwide survey. Dig Dis.

[25] Bruix J, Reig M, Sherman M (2016). Evidence-based diagnosis, staging, and treatment of patients with hepatocellular carcinoma. Gastroenterology.

[26] Giannini EG, Farinati F, Ciccarese F, Pecorelli A, Rapaccini GL, Marco MD, Benvegnù L, Caturelli E, Zoli M, Borzio F (2015). Prognosis of untreated hepatocellular carcinoma. Hepatology.

[27] Dohmen K, Shigematsu H, Irie K, Ishibashi H (2003). Longer survival in female than male with hepatocellular carcinoma. J Gastroenterol Hepatol.

[28] Ladenheim MR, Kim NG, Nguyen P, Le A, Stefanick ML, Garcia G, Nguyen MH (2016). Sex differences in disease presentation, treatment and clinical outcomes of patients with hepatocellular carcinoma: a single-centre cohort study. BMJ Open Gastroenterology.

[29] Capuano G, Daniele B, Gaeta G, Gallo C, Perrone F (1998). A new prognostic system for hepatocellular carcinoma: a retrospective study of 435 patients. Hepatology.

[30] Chevret S, Trinchet J-C, Mathieu D, Rached AA, Beaugrand M, Chastang C (1999). A new prognostic classification for predicting survival in patients with hepatocellular carcinoma. J Hepatol.

[31] Leung TWT, Tang AMY, Zee B, Lau WY, Lai PBS, Leung KL, Lau JTF, Yu SCH, Johnson PJ (2002). Construction of the Chinese University Prognostic Index for hepatocellular carcinoma and comparison with the TNM staging system, the Okuda staging system, and the Cancer of the Liver Italian Program staging system. Cancer.

[32] Burnett NP, Dunki-Jacobs EM, Callender GG, Anderson RJ, Scoggins CR, McMasters KM, Martin RC (2013). Evaluation of alpha-fetoprotein staging system for hepatocellular carcinoma in noncirrhotic patients. Am Surg.

[33] Kinoshita A, Onoda H, Fushiya N, Koike K, Nishino H, Tajiri H (2015). Staging systems for hepatocellular carcinoma: Current status and future perspectives. World J Hepatol.

[34] Subramaniam S, Kelley RK, Venook AP (2013). A review of hepatocellular carcinoma (HCC) staging systems. Chinese Clinical Oncology.

[35] Ma WL, Hsu CL, Yeh CC, Wu MH, Huang CK, Jeng LB, Hung YC, Lin TY, Yeh S, Chang C (2012). Hepatic androgen receptor suppresses hepatocellular carcinoma metastasis through modulation of cell migration and anoikis. Hepatology.

[36] Li Z, Tuteja G, Schug J, Kaestner Klaus H (2012). Foxa1 and Foxa2 are essential for sexual dimorphism in liver cancer. Cell.

[37] Yu MW, Chang HC, Chang SC, Liaw YF, Lin SM, Liu CJ, Lee SD, Lin CL, Chen PJ, Lin SC (2003). Role of reproductive factors in hepatocellular carcinoma: Impact on hepatitis B– and C–related risk. Hepatology.

[38] McGlynn KA, Hagberg K, Chen J, Braunlin M, Graubard BI, Suneja N, Jick S, Sahasrabuddhe VV (2016). Menopausal hormone therapy use and risk of primary liver cancer in the clinical practice research datalink. Int J Cancer.

[39] Hassan MM, Botrus G, Abdel-Wahab R, Wolff RA, Li D, Tweardy D, Phan AT, Hawk E, Javle M, Lee J-S (2017). Estrogen replacement reduces risk and increases survival times of women with hepatocellular carcinoma. Clin Gastroenterol Hepatol.

[40] Nowak A, Findlay M, Culjak G, Stockler M (2004). Tamoxifen for hepatocellular carcinoma. Cochrane Database Syst Rev.

[41] (GRETCH) GdEedTdCHe (2004). Randomized trial of leuprorelin and flutamide in male patients with hepatocellular carcinoma treated with tamoxifen. Hepatology.

[42] Miceli V, Cocciadiferro L, Fregapane M, Zarcone M, Montalto G, Polito LM, Agostara B, Granata OM, Carruba G (2011). Expression of wild-type and variant estrogen receptor alpha in liver carcinogenesis and tumor progression. OMICS.

[43] Villa E, Colantoni A, Camma C, Grottola A, Buttafoco P, Gelmini R, Ferretti I, Manenti F (2003). Estrogen receptor classification for hepatocellular carcinoma: comparison with clinical staging systems. J Clin Oncol.

