# Supplementary material for: Is there a sex difference in postoperative prognosis of hepatocellular carcinoma?
Source: BMC Cancer. 2019 Mar 20;19:250. doi: 10.1186/s12885-019-5453-3 (PMC6425676; doi:10.1186/s12885-019-5453-3)
Supplement: Supplementary file 1 — Table S1. Sex specific 1-, 3-, and 5-year survival rates. The table lists 1-, 3-, and 5-year overall survival, metastasis-free survival, and recurrence-free survival rates in HCC patients of different sexes. (PDF 79 kb) [file 12885_2019_5453_MOESM1_ESM.pdf]

Supplement Table 1. Sex specific 1-, 3-, and 5-year survival rates

		Survival rate		
		Overall	Metastasis-free	Recurrence-free
1-year	Female	94.9%	92.4%	78.8%
	Male	95.0%	91.0%	70.6%
3-year	Female	89.0%	87.3%	62.7%
	Male	91.0%	83.9%	52.3%
5-year	Female	86.4%	84.7%	54.2%
	Male	88.4%	82.2%	46.5%
