# Supplementary material for: Is there a sex difference in postoperative prognosis of hepatocellular carcinoma?
Source: BMC Cancer. 2019 Mar 20;19:250. doi: 10.1186/s12885-019-5453-3 (PMC6425676; doi:10.1186/s12885-019-5453-3)
Supplement: Supplementary file 2 — Table S2. Clinicopathological factors associated with intrahepatic recurrence free survival in male and female HCC. The table lists univariate and multivariate analysis of clinicopathological factors associated with intrahepatic recurrence-free survival in HCC patients of different sexes. (PDF 91 kb) [file 12885_2019_5453_MOESM2_ESM.pdf]

Supplement Table 2. Clinicopathological factors associated with intrahepatic recurrence free survival in male and female HCC

	Male						Female					
	Univariate			Multivariate			Univariate			Multivariate		
	HR	95% CI	P	HR	95% CI	P	HR	95% CI	P	HR	95% CI	P
Age, per year increase	1	0.991 - 1.010	0.978				1.013	0.994 - 1.032	0.191			
Anti-HCV, positive = 1	1.072	0.783 - 1.467	0.664				1.195	0.712 - 2.006	0.501			
HBsAg, positive = 1	0.88	0.655 - 1.182	0.395				1.182	0.701 - 1.992	0.531			
Liver cirrhosis, Yes = 1	1.019	0.782 - 1.327	0.89				1.479	0.867 - 2.523	0.151			
Microvascular invasion, Yes = 1	2.366	1.815 - 3.084	< **0.001**	2.143	1.636 - 2.806	< **0.001**	2.226	1.246 - 3.977	**0.007**	2.528	1.399 - 4.567	**0.002**
Macrovascular invasion, Yes = 1	1.435	0.975 - 2.110	0.067				1.518	0.738 - 3.121	0.256			
Histology, per grade increase	1.208	1.002 - 1.456	**0.047**				1.207	0.815 - 1.786	0.347			
Capsule, Yes = 1	0.999	0.746 - 1.337	0.994				1.081	0.608 - 1.921	0.79			
Tumor number, per number increase	1.178	1.050 - 1.321	**0.005**	1.13	1.006 - 1.270	**0.039**	1.194	0.890 - 1.602	0.237			
Ascites, Yes = 1	2.137	1.359 - 3.361	**0.001**				0.367	0.089 - 1.505	0.164			
Alcoholism, Yes = 1	1.116	0.849 - 1.467	0.432				1.868	0.454 - 7.683	0.387			
Largest tumor size, per cm increase	1.012	0.997 - 1.027	0.129				1.075	1.015 - 1.139	**0.014**			
AFP, per 1000 ng/mL increase	1.004	1.000 - 1.008	0.082				1.002	0.999 - 1.005	0.134			
Albumin, per g/L increase	0.631	0.502 - 0.791	< **0.001**	0.707	0.558 - 0.896	**0.004**	0.729	0.453 - 1.171	0.191			
Bilirubin, per mg/dL increase	0.94	0.821 - 1.076	0.367				1.412	0.835 - 2.389	0.198			

Prothrombin time, per sec increase	1.078	0.995 - 1.168	0.066				1.074	0.902 - 1.279	0.424			
Creatinine, per mg/dL increase	0.939	0.773 - 1.141	0.527				0.833	0.599 - 1.158	0.277			
AST, per U/L increase	1.002	1.001 - 1.003	< **0.001**	1.002	1.000 - 1.003	**0.016**	1.004	1.002 - 1.007	**0.001**	1.005	1.002 - 1.007	< **0.001**
ALT, per U/L increase	1.001	1.000 - 1.002	**0.025**				1.004	1.000 - 1.009	0.074			

Multivariate analysis was performed using stepwise forward mode.
