# Supplementary material for: Is there a sex difference in postoperative prognosis of hepatocellular carcinoma?
Source: BMC Cancer. 2019 Mar 20;19:250. doi: 10.1186/s12885-019-5453-3 (PMC6425676; doi:10.1186/s12885-019-5453-3)
Supplement: Supplementary file 3 — Table S3. Clinicopathological factors associated with distant metastasis free survival in male and female HCC. The table lists univariate and multivariate analysis of clinicopathological factors associated with distant metastasis free survival in HCC patients of different sexes, (PDF 91 kb) [file 12885_2019_5453_MOESM3_ESM.pdf]

Supplement Table 3. Clinicopathological factors associated with distant metastasis free survival in male and female HCC.

	Male						Female					
	Univariate			Multivariate			Univariate			Multivariate		
	HR	95% CI	P	HR	95% CI	P	HR	95% CI	P			
Age, per year increase	0.997	0.982 - 1.013	0.731				0.972	0.944 - 1.001	0.059			
Anti-HCV, positive = 1	0.822	0.469 - 1.443	0.496				0.75	0.285 - 1.974	0.56			
HBsAg, positive = 1	0.777	0.477 - 1.267	0.312				1.613	0.613 - 4.245	0.333			
Liver cirrhosis, Yes = 1	0.809	0.521 - 1.256	0.345				0.732	0.297 - 1.803	0.498			
Microvascular invasion, Yes = 1	3.048	1.955 - 4.750	< **0.001**	2.87	1.838 - 4.482	< **0.001**	2.246	0.852 - 5.924	0.102			
Macrovascular invasion, Yes = 1	2.583	1.506 - 4.433	**0.001**	2.405	1.396 - 4.143	**0.002**	2.599	0.861 - 7.844	0.09			
Histology, per grade increase	1.302	0.948 - 1.788	0.103				1.491	0.732 - 3.039	0.271			
Capsule, Yes = 1	0.956	0.589 - 1.552	0.856				0.613	0.241 - 1.558	0.304			
Tumor number, per number increase	1.285	1.069 - 1.543	**0.008**				1.278	0.774 - 2.111	0.337			
Ascites, Yes = 1	0.867	0.316 - 2.378	0.782				0.044	0 - 88.219	0.421			
Alcoholism, Yes = 1	1.099	0.695 - 1.737	0.686				0.048	0 - 15946.169	0.639			
Largest tumor size, per cm increase	1.025	1.006 - 1.045	**0.01**				1.13	1.032 - 1.238	**0.008**			
AFP, per 1000 ng/mL increase	1.007	1.001 - 1.013	**0.017**	1.007	1.001 - 1.013	**0.032**	1.005	1.001 - 1.008	**0.004**	1.005	1.002 - 1.008	**0.002**

Albumin, per g/L increase	0.711	0.480 - 1.053	0.089	0.97	0.406 - 2.321	0.946			
Bilirubin, per mg/dL increase	0.854	0.611 - 1.194	0.355	0.782	0.186 - 3.291	0.738			
Prothrombin time, per sec increase	1.068	0.930 - 1.226	0.351	0.909	0.629 - 1.315	0.613			
Creatinine, per mg/dL increase	0.508	0.194 - 1.332	0.169	0.083	0.005 - 1.296	0.076			
AST, per U/L increase	1.001	0.999 - 1.003	0.51	1.005	1.002 - 1.009	**0.002**	1.006	1.002 - 1.009	**0.001**
ALT, per U/L increase	0.999	0.996 -1.002	0.394	1	0.991 - 1.009	0.997			

Multivariate analysis was performed using stepwise forward mode.
