# Supplementary material for: Is there a sex difference in postoperative prognosis of hepatocellular carcinoma?
Source: BMC Cancer. 2019 Mar 20;19:250. doi: 10.1186/s12885-019-5453-3 (PMC6425676; doi:10.1186/s12885-019-5453-3)
Supplement: Supplementary file 4 — Table S4. Clinicopathological factors associated with overall survival in male and female HCC. The table lists univariate and multivariate analysis of clinicopathological factors associated with overall survival in HCC patients of different sexes. (PDF 91 kb) [file 12885_2019_5453_MOESM4_ESM.pdf]

Supplement Table 4. Clinicopathological factors associated with overall survival in male and female HCC.

	Male						Female					
	Univariate			Multivariate			Univariate			Multivariate		
	HR	95% CI	P	HR	95% CI	P	HR	95% CI	P	HR	95% CI	P
Age, per year increase	0.986	0.996 - 1.006	0.162				1.03	0.991 - 1.070	0.133			
Anti-HCV, positive = 1	0.782	0.376 - 1.614	0.505				0.878	0.328 - 2.349	0.796			
HBsAg, positive = 1	1.139	0.581 - 2.235	0.704				0.476	0.186 - 1.223	0.123			
Liver cirrhosis, Yes = 1	0.701	0.400 - 1.228	0.215				1.718	0.611 - 4.831	0.305			
Microvascular invasion, Yes = 1	1.941	1.098 - 3.433	**0.023**				2.017	0.753 - 5.400	0.163			
Macrovascular invasion, Yes = 1	2.193	1.060 - 4.539	**0.034**				2.371	0.766 - 7.339	0.134			
Histology, per grade increase	1.339	0.894 - 2.006	0.157				1.293	0.626 - 2.670	0.488			
Capsule, Yes = 1	0.931	0.501 - 1.731	0.821				0.609	0.225 - 1.648	0.329			
Tumor number, per number increase	1.304	1.046 - 1.627	**0.018**	1.316	1.046 - 1.656	**0.019**	0.989	0.545 - 1.794	0.972			
Ascites, Yes = 1	3.16	1.474 - 6.772	**0.003**	2.155	0.956 - 4.855	0.064	2.612	0.753 - 9.060	0.13			
Alcoholism, Yes = 1	1.375	0.777 - 2.436	0.274				0.048	0 - 78121.465	0.677			
Largest tumor size, per cm increase	1.026	1.002 - 1.050	**0.033**				1.024	0.919 - 1.141	0.665			
AFP, per 1000 ng/mL increase	1.005	0.996 - 1.014	0.313				1.001	0.996 - 1.005	0.73			

Albumin, per g/L increase	0.495	0.313 - 0.783	**0.003**	0.602	0.366 - 0.989	**0.045**	0.424	0.189 - 0.955	**0.038**			
Bilirubin, per mg/dL increase	1.081	0.941 - 1.242	0.273				1.844	1.177 - 2.891	**0.008**	1.844	1.177 - 2.891	**0.008**
Prothrombin time, per sec increase	1.142	0.975 - 1.337	0.1				0.842	0.562 - 1.262	0.406			
Creatinine, per mg/dL increase	0.947	0.673 - 1.334	0.757				1.012	0.743 - 1.378	0.94			
AST, per U/L increase	1.002	1.001 - 1.004	**0.009**	1.002	1.000 - 1.004	0.052	1.002	0.996 - 1.008	0.538			
ALT, per U/L increase	1	0.998 - 1.002	0.978				1.001	0.992 - 1.009	0.866			

Multivariate analysis was performed using stepwise forward mode.
